# Trajectories of primary health care utilization: a 10-year follow-up after the Swedish Patient Choice Reform of primary health care

**DOI:** 10.1186/s12913-023-10326-9

**Published:** 2023-11-23

**Authors:** Hannes Kohnke, Andrzej Zielinski, Anders Beckman, Henrik Ohlsson

**Affiliations:** 1 Department of Clinical Sciences, Faculty of Medicine, Lund University, Lund, Sweden; 2 Bräkne-Hoby Vårdcentral, Parkvägen 4, Bräkne-Hoby, 370 10 Sweden; 3 Blekinge Centre of Competence, Region Blekinge, Vårdskolevägen 5, Karlskrona, 371 41 Sweden; 4 Department of Clinical Sciences, Center for Primary Health Care Research, Lund University Clinical Research Centre (CRC), Box 50332, Malmö, 202 13 Sweden

**Keywords:** Primary health care, Delivery of health care, Health care utilization, Health equity, Health policy, Patient choice, Privatization, Sweden

## Abstract

**Background:**

In January 2010, the choice reform was instituted in Swedish primary health care establishing free entry for private primary health care providers and enabling patients to choose freely among primary health care centers. The motivation behind the reform was to improve access to primary care and responsiveness to patient expectations. Reform effects on health care utilization have previously been investigated by using subgroup analyses assuming a pattern of homogeneous subgroups of the population. By using a different methodological approach, the aim of this study was to, from an equity perspective, investigate long term trends of primary health care utilization following the choice reform.

**Method:**

A closed cohort was created based on register data from Region Skåne, the third most populated region in Sweden, describing individuals’ health care utilization between 2007–2017. Using a novel approach, utilization data, measured as primary health care visits, was matched with socioeconomic and geographic determinants, and analyzed using logistic regression models.

**Results:**

A total of 659,298 individuals were included in the cohort. Sex differences in utilization were recorded to decrease in the older age group and to increase in the younger age group. Multivariable logistic regression showed increasing utilization in older men to be associated with higher socioeconomic position, while in women it was associated with lower socioeconomic position. Furthermore, groups of becoming high utilizers were all associated with lower socioeconomic position and with residence in urban areas.

**Conclusion:**

The impact of demographic, socioeconomic and geographic determinants on primary health care utilization varies in magnitude and direction between groups of the population. As a result, the increase in utilization as observed in the general population following the choice reform is unevenly distributed between different population groups.

**Supplementary Information:**

The online version contains supplementary material available at 10.1186/s12913-023-10326-9.

## Background

In Sweden, as in many countries, primary health care (PHC) forms the basis of the health care system and is regarded as an efficient way to address main causes of, and risk factors for, poor health [1–3]. In most societies, PHC is distinguished as the part of the health care system that benefits those of lower socioeconomic status (SES) to a higher degree [4, 5]. Fundamental for providing an equitable PHC is that physicians in PHC (general practitioners or GPs) appropriate their services according to differences in patient needs. The appropriation of GP utilization is in turn influenced by several other factors such as geographical inequity in the distribution of primary health care centers (PCCs), user fees, health literacy and incentives related to funding and competition for patients [6].

In Sweden, the health care system is mainly tax-funded and citizens are ensured a universal coverage with no or minimal patient fees. PHC accounts for about 20% of total health care expenditures and is provided by multi-professional PCCs staffed with nurses, general practitioners, and to varying extent, other health care professionals [7]. Half of all doctor visits are made in PHC [7]. Twenty one independent regions are responsible for the financing and organization of health care. Although PHC in a majority of the regions has no formal gatekeeping function, typically, PCCs serve as patients’ entry point into the health care system and as the continuing focal point for most needed health care services [3]. This study is set in Region Skåne, the third largest Swedish region with 1.3 million residents.

Dissatisfaction with the performance of PHC services has led to political interest in policies that promote competition in many countries [8]. Quasi-markets and public competition have been introduced and assessed in health care systems around the world since the early 1990s, with varying degrees of success [9]. Between 2007 and 2010 a wave of PHC reforms was initiated throughout Sweden. These reforms, commonly referred to as the *Patient Choice Reform*, established free entry for private PHC providers and enabled patients to choose freely among PCCs. The motivation behind the reforms was to improve access to PHC and responsiveness to patient expectations [10]. Following the reform date in Region Skåne (1 May 2009), any provider that fulfilled the specified conditions for accreditation would be allowed to open a PCC, it would no longer be allowed for providers to reject patients who wished to enroll, and patients could choose freely among all PCCs in the region. Before the reform, patient choice was more limited, and providers were allowed to reject patients living outside their geographical catchment area. Also, with the reform the reimbursement system for PCCs was gradually redesigned from being based 80% on capitation to enrolled patients and 20% on fee-for-service, to being solely capitation-based [11]. To accommodate for variations in expected costs, capitation is higher for patients with many diagnoses or low SES. In 2010, it became mandatory for all regions to provide freedom of establishment for private primary care providers, and patients with choice of provider, through a change in the national Health Care Act [12]. Following years, the patient choice system in Region Skåne gradually became more comprehensive, specifying standards for coordination of elderly care and various diagnose specific nurse led clinics, and also came to include certain specialized health care services [11].

The effects of the Patient Choice Reform have been analyzed in scientific reports and by government and regional agencies. Overall, the number of PCCs has increased and new PCCs have mainly been established in urban areas or in areas where healthcare needs are lower [13–15]. The closing of PCCs has mainly affected areas with higher expected health care needs [13]. Consequently, concerns have been raised about increasing geographical inequity in PCC allocation along with declining access and continuity of care for part of the population [13, 16, 17]. With an increasing number and broader range of providers, the health care system has become more complex and challenging to navigate. Following this, demands on patient participation and health literacy are expected to increase – health literacy referring to individuals’ abilities to access, understand and communicate health-related information needed to make informed health decisions [18]. Investigations of the impact of the reform show an overall increase in PHC utilization but individuals with higher income or minor symptoms have increased their utilization to a higher degree than those with low income or more severely ill [6, 13, 19]. However, these investigations are in part limited by cross-sectional designs, not being able to detect changes of utilization on an individual level, or short follow-up periods after the reform.

A common approach when assessing the effects of the Patient Choice Reform on health care utilization has been by subgroup analyses assuming a pattern of homogeneous subgroups of the population, each subgroup having similar outcomes [6, 13, 19–21]. However, such characteristics may not be true in gross subgroup divisions where outcomes may not work the same way in all patients. The use of a person-oriented technique for subgrouping, that does not assume the relationship between variables to be the same for all patients, can provide an understanding of how differences in independent variables between patients affect outcome [22]. To our knowledge such an approach has never been applied when investigating effects of the Patient Choice Reform. By using a method allowing for person-oriented subgroup analyses, the purpose of this study was to investigate long term trends of PHC utilization following the Patient Choice Reform, and to identify groups of the population with changed utilization.

## Methods

This study is based on administrative register data from Region Skåne, describing individual health care utilization of all publicly funded PHC services. Privately funded health care services, which comprise less than 1% of total health care spendings in Sweden, were not included [23]. On the individual level, health care utilization data from Region Skåne was linked to demographic (age and sex), socioeconomic, (income, education and civil status) and geographic (municipality of residence) determinants obtained from Statistics Sweden.

### Population

Retrospectively, a closed cohort was created of all inhabitants with a registered address in Region Skåne between 2007 and 2017. Individuals aged under 20 or over 69 in 2007 or have had a registered address outside of Region Skåne at some point during follow-up were excluded from the study. The lower age limit was chosen to allow the use of income and educational level as a proxy for SES, and the upper limit to allow inclusion of retirees but to exclude older age groups with a tapering population.

### Outcome measure

The outcome measure was defined as the total number of GP visits per individual and calendar year. GP visits due to preventative health care (i.e. maternity care, child health care) were excluded. Based on frequency analyses of the number of annual visits, four distinct utilization-groups were created; 0 visits, 1 visit, 2–3 visits, and more than 3 visits. These groups will be referred to as low- (0–1 visit), intermediate- (2–3 visits), and high-utilizers (> 3 visits).

### Independent variables

All independent variables (age, sex, income, educational level, civil status and municipality of residence) were defined at baseline (1 January 2007).

Due to the influence of age on income, analyses were made on three different age groups based on age at baseline; 20–34 (young), 35–54 (middle-aged) and 55–69 (older) years of age (corresponding birth years: 1987-73, 1972-53, and 1952-38).

Income was defined as pre-tax household income equalized to the number of family members. Pre-tax income included earnings from employment, business, income transfers (e.g., pension payments, unemployment benefits, or paid sick leave), and capital gain, but not return of capital. The income variable was categorized into three equal-sized groups.

Educational level was categorized to elementary school, high school or higher education, and civil status to either married/cohabiting or single/divorced/widow/widower.

The level of urbanicity of municipality of residence was grouped into three categories. The Swedish Association of Local Authorities and Regions classifies municipalities in nine categories based on structural parameters such as population, degree of urbanization and commuting patterns [24]. This classification was modified to fit into three groups: 1) large cities (population over 200,000) and medium-sized towns (population over 50,000), 2) small towns (population over 15,000) and commuting municipalities near large cities, 3) rural municipalities and commuting municipalities near medium- and small-sized towns. These three groups are referred to as urban, semi-urban and rural.

### Statistical method

Datasets were constructed for each sex and age group. Separately for each dataset, group-based trajectory modeling (GBTM) was used to identify individuals with similar health care utilization patterns. GBTM is a semi-parametric model, designed to analyze longitudinal data [25, 26]. Based on the assumption of a discrete distribution of the population, GBTM makes it possible to distinguish subgroups of individuals in a population with a similar trajectory [22, 25, 26]. For each individual, the model determines the probability of belonging to one subgroup or another (posterior group probability). Individuals are assigned to a subgroup based on their highest posterior group probability [25, 26].

In each dataset the number of trajectory-groups, indicated by the observed variables, was determined by comparing model fit statistics between nested models. Improvement in model fit was indicated by smaller values of the Bayesian information criterion (BIC) and Akaike’s Information Criterion (AIC). However, as the number of trajectory-groups is influenced by the number of observed variables, both empirical (improved model fit) and theoretical (model interpretability) aspects were considered. Given the large sample size, statistical power allowed for identification of trajectory-groups too small to be useful in clinical- or research work. Hence, an arbitrary limit was set and only datasets where all identified trajectory-groups had a prevalence of ≥ 2% of the dataset population were considered for further analyses.

In the next step, trajectory-groups with similar health care utilization at baseline and with diverging patterns over time, were selected pairwise. Logistic regression was used to describe differences between the pairwise selected trajectory-groups (see Tables 2 and 3). Both bivariate- and multivariable regression models were constructed, the latter with age, income, education, civil status, and municipality of residence as independent variables. In the regression models, age was treated as a continuous variable and all other as categorical. The results were presented as odds ratios (ORs) with 95% confidence intervals (CIs).

GBTMs were performed with SAS 9.4 (SAS Institute, Inc., Cary, NC) and logistic regressions with IBM SPSS version 27 (IBM Corp., Armonk, NY).

## Results

### Descriptive

In 2007, Region Skåne had 766,029 residents aged 20 to 69 where of 659,298 (86%) were included in the study. Study population demographics are provided in Table 1.

**Table 1:** Cohort population characteristics and general trends in utilization in defined sex- and age groups between 2007 and 2017

**Sex**		**Male**			**Female**			
**Age group (years)**		**20–34**	**35–54**	**55–69**	**20–34**	**35–54**	**55–69**	**Total**
**Number of individuals (N)**		93,427	145,045	88,365	92,405	145,484	94,572	659,298
**Income (% of total in age group)**	low income	30.5	31.8	29.8	38.9	36.0	36.6	34.0
	medium income	30.3	33.4	34.0	35.1	32.7	32.7	33.0
	high income	39.2	34.8	36.2	26.0	31.3	30.7	33.0
**Education (% of total in age group)**	primary school	4.9	6.2	16.3	4.0	4.2	17.1	8.2
	secondary school	39.0	39.6	42.0	35.8	38.9	43.2	39.7
	higher education	56.1	54.2	41.6	60.3	57.0	39.7	52.2
**Civil status (% of total in age group)**	single	81.1	47.0	32.9	72.0	44.3	38.0	51.5
	married/cohabitant	18.9	53.0	67.1	28.0	55.7	62.0	48.5
**Municipality of residence (% of total in age group)**	urban	50.5	40.6	36.1	51.1	40.8	38.0	42.6
	semi-urban	25.0	30.5	31.9	25.0	30.5	31.4	29.3
	rural	24.5	28.9	32.0	23.9	28.7	30.5	28.2
**Mean number of annual GP visits per person**	2007	0.71	0.93	1.32	1.18	1.4	1.73	1.21
	2012	0.83	1.05	1.58	1.48	1.58	1.95	1.41
	2017	0.8	1.11	1.66	1.41	1.6	1.94	1.42
**Absolute increase in annual GP visits 2007 vs, 2017 (N)**		6,657	26,369	29,579	19,017	29,585	20,163	131,370
**Relative increase in annual GP visits 2007 vs, 2017 (%)**		11	20	25	20	15	12	17

### Visits to GPs

In total, 9,577,498 GP visits were registered during the study period. Of these visits, 60% of were made by women and 3% of the study population made no visits at all. The average number of annual visits per individual rose from 1.21 in 2007 to 1.42 in 2017, corresponding to an absolute increase by 131,370 visits or relative increase by 17% (Table 1). The increase in utilization varied between groups but was for all groups more distinct between 2007–2012 and gender specific differences were most pronounced in the youngest and oldest age groups. Compared to the corresponding age group of the opposite sex, the relative increase in utilization was 9 percentage points higher for younger women and 13 percentage points higher for older men. Older men had the highest relative increase (25%), but nonetheless fewer annual GP visits on average than the corresponding female age group in 2017 (1.66 and 1.94 respectively).

### Trajectories of primary health care utilization

**Fig. 1:**
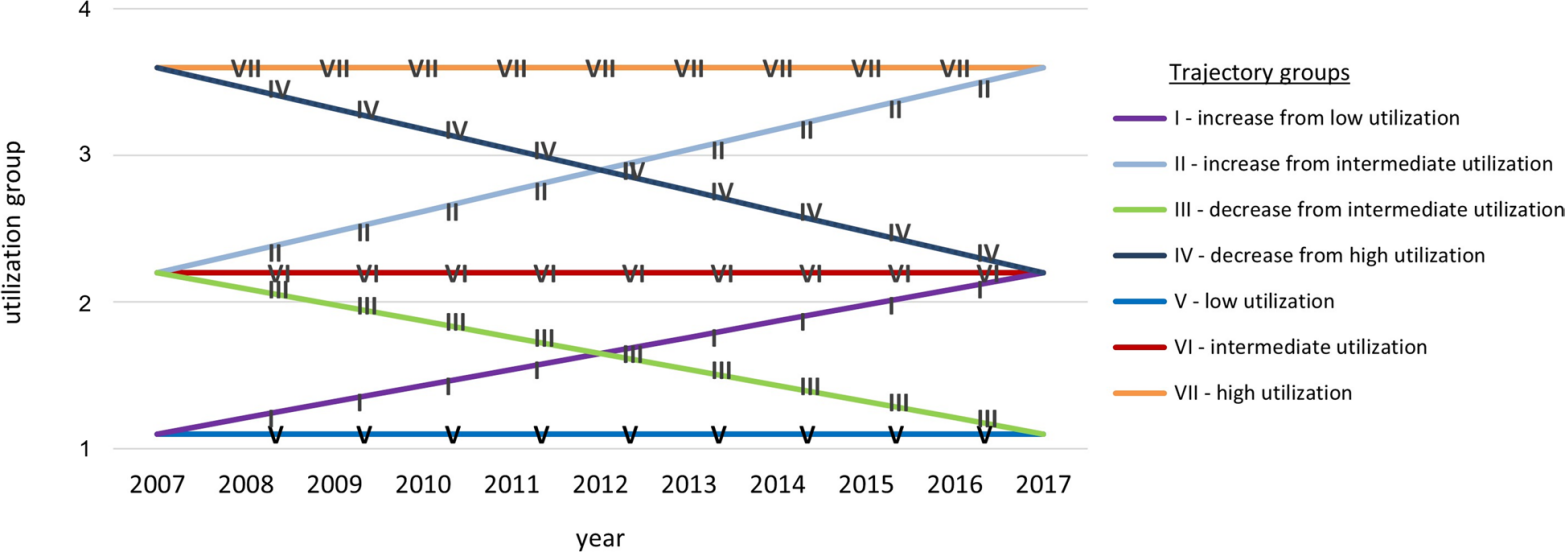
Generic trajectory analysis output. Generic trajectory analysis output. On the Y-axis, the number of annual GP visits per individual are categorized by 4 utilization-groups corresponding to; 0 visits, 1 visit, 2–3 visits, and more than 3 visits

Based on model fit statistics (see Additional Table 1) six trajectory datasets were chosen for further analysis, one set for each sex- and age group (see Additional Fig. 1). Independent variables varied between trajectory-groups in each dataset (see Additional Table 2a for males and b for females). In general, each dataset followed a generic pattern of trajectories as shown in Fig. 1. Common for each dataset was the presence of trajectory-groups with relatively unchanged continuously low, intermediate, and high utilization as well as trajectories with changing utilization in close to linear fashions. For simplicity, trajectories will henceforth be referred to by their roman numeral as denoted in Fig. 1.

Common for all trajectory datasets, trajectory-groups between low utilization and intermediate utilization (I, III, V and VI) contained larger proportions of individuals. Lowest proportion of individuals were found in trajectory-groups indicating continuously high utilization (VII) or changing utilization between intermediate- and high levels (II and IV). Trajectory-groups with changed utilization between low and intermediate levels were recorded in all sex- and age groups except for in older women. Trajectory-groups with changed utilization between intermediate- and high levels (from intermediate- to high- or from high- to intermediate levels) were exclusively recorded in female groups.

In the trajectory dataset for younger women, trajectories varied from the generic pattern in Fig. 1 by being more dispersed on the y-axis at the starting point, making analysis more complex. Depending on the position of the reference trajectory, above or below the trajectory being analyzed, the results might be subjected to under- or overestimation. Two trajectories showed increased utilization – increase from low utilization (I) and increase from intermediate utilization (II). Due to their positioning relative to reference trajectories, each increasing trajectory was analyzed with two different reference trajectories separately.

### Logistic regression

Results from the bivariate regression model (see Additional Table 3a for males and b for females) differed inconsiderably from the multivariable model (Tables 2 for males and 3 for females).

**Table 2:** Multivariable logistic regression on male trajectory-groups showing odds ratios (ORs) of change in utilization due to differences in predisposing and enabling factors

**Age group (years)**		**20–34**		**35–54**		**55–69**	
**Compared trajectory-groups**^**a**^		**Group 5 vs. 1 (ref.)**	**Group 3 vs. 2 (ref.)**	**Group 5 vs. 3 (ref.)**	**Group 2 vs. 4 (ref.)**	**Group 4 vs. 1 (ref.)**	**Group 2 vs. 3 (ref.)**
**Corresponding generic trajectory-groups**^**b**^		**I vs. V (ref.)**	**III vs. VI (ref.)**	**I vs. V (ref.)**	**III vs. VI (ref.)**	**I vs. V (ref.)**	**III vs. VI (ref.)**
**Analyzed utilization change**		**OR (CI) for increase from low utilization**	**OR (CI) for decrease from intermediate utilization**	**OR (CI) for increase from low utilization**	**OR (CI) for decrease from intermediate utilization**	**OR (CI) for increase from low utilization**	**OR (CI) for decrease from intermediate utilization**
**Age**		1.01 (1.00–1.01)	0.98 (0.97–0.98)	1.05 (1.04–1.05)	0.95 (0.94–0.95)	1.04 (1.03–1.05)	0.93 (0.92–0.93)
**Income**	low income	1	1	1	1	1	1
	middle income	1.04 (0.98–1.09)	1.13 (1.08–1.18)	0.98 (0.94–1.02)	1.21 (1.17–1.26)	1.33 (1.24–1.43)	0.87 (0.82–0.92)
	high income	0.98 (0.94–1.03)	1.27 (1.21–1.32)	0.88 (0.84–0.92)	1.34 (1.29–1.39)	1.27 (1.18–1.37)	0.87 (0.82–0.92)
**Education**	primary school	1	1	1	1	1	1
	secondary school	0.94 (0.85–1.04)	1.27 (1.16–1.38)	0.92 (0.86–0.99)	1.11 (1.03–1.18)	1.08 (0.99–1.17)	0.94 (0.88–1.01)
	higher education	0.75 (0.68–0.83)	1.67 (1.53–1.81)	0.78 (0.73–0.84)	1.42 (1.32–1.52)	0.89 (0.81–0.97)	1.04 (0.97–1.11)
**Civil status**	single	1	1	1	1	1	1
	married or cohabitant	1.16 (1.10–1.23)	0.91 (0.86–0.95)	1.03 (1.00–1.07)	1 (0.96–1.03)	1.27 (1.20–1.35)	0.85 (0.81–0.89)
**Municipality of residence**	urban	1	1	1	1	1	1
	semi-urban	1.01 (0.96–1.07)	1.00 (0.95–1.04)	1.02 (0.98–1.06)	1.04 (1–1.08)	1.05 (0.98–1.12)	0.92 (0.87–0.97)
	rural	0.96 (0.91–1.01)	1.04 (0.99–1.09)	0.99 (0.95–1.03)	1.14 (1.1–1.19)	1.14 (1.07–1.22)	1.09 (1.03–1.15)

*OR* Odds ratio, *CI* Confidence interval

^a^As shown in Additional Fig. 1

^b^As shown in Fig. 1

**Table 3:** Multivariable logistic regression on female trajectory-groups showing odds ratios (ORs) of change in utilization due to differences in predisposing and enabling factors

**Age group (years)**		**20–34**				**35–54**				**55–69**	
**Compared trajectory-groups**^**a**^		**Group 5 vs. 1 (ref.)**	**Group 5 vs. 3 (ref.)**	**Group 2 vs. 3 (ref.)**	**Group 2 vs. 4 (ref.)**	**Group 3 vs. 1 (ref.)**	**Group 5 vs. 2 (ref.)**	**Group 6 vs. 7 (ref.)**	**Group 4 vs. 2 (ref.)**	**Group 5 vs. 4 (ref.)**	**Group 3 vs. 6 (ref.)**
**Corresponding generic trajectory-groups**^**b**^		**II vs. VI (ref.)**	**II vs. VI (ref.)**	**II vs. VI (ref.)**	**II vs. VI (ref.)**	**I vs. V (ref.)**	**II vs. VI (ref.)**	**IV vs. VII (ref.)**	**III vs. VI (ref.)**	**II vs. VI (ref.)**	**IV vs. VII (ref.)**
**Analyzed utilization change**		**OR (CI) for increase from low utilization**	**OR (CI) for increase from low utilization**	**OR (CI) for increase from intermediate utilization**	**OR (CI) for increase from intermediate utilization**	**OR (CI) for increase from low utilization**	**OR (CI) for increase from intermediate utilization**	**OR (CI) for decrease from high utilization**	**OR (CI) for decrease from intermediate utilization**	**OR (CI) for increase from intermediate utilization**	**OR (CI) for decrease from high utilization**
**Age**		0.99 (0.98–0.99)	0.98 (0.98–0.99)	1.00 (1.00–1.01)	1.00 (0.99–1.00)	1.01 (1.01–1.01)	1.01 (1.01–1.02)	0.99 (0.99–1.00)	0.96 (0.96–0.96)	1.04 (1.04–1.05)	0.97 (0.96–0.98)
**Income**	low income	1	1	1	1	1	1	1	1	1	1
	middle income	1.06 (1.01–1.12)	0.88 (0.84–0.92)	0.70 (0.66–0.76)	0.70 (0.65–0.75)	1.01 (0.96–1.06)	0.73 (0.69–0.77)	1.36 (1.26–1.47)	1.13 (1.09–1.18)	0.89 (0.83–0.95)	1.26 (1.16–1.37)
	high income	0.95 (0.90–1.00)	0.86 (0.82–0.90)	0.53 (0.48–0.57)	0.65 (0.60–0.71)	0.93 (0.88–0.98)	0.59 (0.56–0.63)	1.58 (1.44–1.73)	1.30 (1.25–1.36)	0.85 (0.79–0.92)	1.35 (1.22–1.48)
**Education**	primary school	1	1	1	1	1	1	1	1	1	1
	secondary school	0.92 (0.81–1.04)	0.82 (0.73–0.92)	0.63 (0.55–0.73)	0.81 (0.71–0.92)	1.21 (1.08–1.35)	0.89 (0.80–0.99)	1.25 (1.10–1.42)	1.07 (0.98–1.17)	1.05 (0.97–1.14)	1.04 (0.95–1.13)
	higher education	0.72 (0.64–0.82)	0.79 (0.71–0.88)	0.36 (0.31–0.41)	0.65 (0.57–0.74)	1.05 (0.94–1.17)	0.69 (0.62–0.77)	1.56 (1.37–1.78)	1.27 (1.16–1.38)	0.99 (0.91–1.08)	1.21 (1.09–1.34)
**Civil status**	single	1	1	1	1	1	1	1	1	1	1
	married or cohabitant	1.13 (1.08–1.19)	1.03 (0.99–1.08)	1.24 (1.15–1.33)	1.11 (1.04–1.19)	1.04 (1.00–1.09)	1.05 (1.00–1.10)	0.93 (0.87–1.00)	0.98 (0.95–1.02)	0.91 (0.85–0.96)	1.04 (0.97–1.12)
**Municipality of residence**	urban	1	1	1	1	1	1	1	1	1	1
	semi-urban	0.99 (0.94–1.04)	0.86 (0.82–0.90)	0.90 (0.83–0.97)	0.84 (0.78–0.90)	1.02 (0.97–1.07)	0.89 (0.84–0.94)	1.22 (1.13–1.32)	1.01 (0.97–1.05)	0.89 (0.84–0.96)	1.07 (0.98–1.16)
	rural	0.93 (0.88–0.98)	0.86 (0.82–0.91)	0.83 (0.76–0.89)	0.88 (0.82–0.95)	1.01 (0.96–1.06)	0.86 (0.81–0.91)	1.22 (1.12–1.32)	1.10 (1.06–1.14)	0.86 (0.81–0.92)	1.08 (0.99–1.17)

*OR* Odds ratio, *CI* Confidence interval

^a^As shown in Additional Fig. 1

^b^As shown in Fig. 1

Multivariable regression analyses showed consistent effects of age on utilization. In most trajectory-groups, increased utilization was associated with increasing age, and decreased utilization was associated with decreasing age. These associations were stronger among older men and women as well as among middle-aged men.

Analyses of the effect of income or education on changed utilization between low- and intermediate levels (I, III) showed distinct results for older men compared to other sex- and age groups where similar change in utilization was recorded. Among older men, ORs for increased utilization from low levels (I) was 1.33 (CI 1.24–1.43) and 1.27 (CI 1.18–1.37) respectively for middle and high income vs. low income. Among middle-aged men and younger women, increased utilization from low levels (I) was associated with lower income and lower education. The strongest association was observed among younger women with recorded ORs of 0.86 (CI 0.82–0.90) and 0.95 (CI 0.90–1.00) for high income vs. low income and 0.79 (CI 0.71–0.88) and 0.72 (CI 0.64-0.082) for higher education vs. primary school). Decreased utilization from intermediate levels (III) was among older men associated with lower income and with a corresponding OR of 0.87 (CI 0.82–0.92) for middle or and high income vs. low income. Among middle-aged and young men and women, the same decrease in utilization was associated with higher income and higher education.

The effect of income or education on changed utilization between intermediate- and high levels (II, IV) showed a similar pattern in all female groups. In all female age groups, increased utilization from intermediate levels (II) was associated with lower education. Among middle-aged and younger women, increased utilization from intermediate levels (II) was also associated with lower income. The strongest associations with low education and low income were recorded among younger women with ORs of 0.65 (CI 0.60–0.71) and 0.53 (0.48–0.57) for high income vs. low income and 0.65 (CI 0.57–0.74) and 0.36 (CI 0.31–0.41) for higher education vs. primary school. Decreased utilization from high levels (IV) was recorded among older and middle-aged women and was here associated with both higher income and education.

Residence in urban areas showed to be indicative of increased utilization when compared to residence in semi-urban or rural areas. When compared to residence in urban areas, differences in the effect on change in utilization was small between residence in semi-urban and rural areas. Without exceptions, all trajectories with increasing utilization from intermediate levels (II) were associated with residence in urban areas. For younger women, increased utilization from both low- and intermediate levels (I and II) were associated with residence in urban areas.

## Discussion

From an equity perspective, this 10-year follow-up examined trends of PHC utilization following the Swedish Patient Choice Reform. Prior investigations of the effects of the reform have in part been limited by design or short follow-up time and by using a novel person-oriented approach to subgrouping, this study adds knowledge to previous findings. First, our findings showed a gradual increase in utilization in the population, unevenly distributed dependent on sex and age. Secondly, socioeconomic determinants affected utilization in different directions in different subgroups. Increasing utilization among older men was associated with higher SES, while among women a similar change was associated with lower SES. Third, increase to high levels of utilization was primarily observed among women, and was irrespective of age associated with lower SES and residence in urban areas, the associations being stronger in younger age groups.

On a national level, PHC utilization in terms of GP visits, has steadily increased until 2011 whereafter utilization has been more constant [13]. In Region Skåne, previous reports have shown the number of GP visits to be relatively constant the years before the reform, to increase the year of the reform, and then to slightly decline between 2011 and 2014 [6, 11]. Similarly, our results showed a more pronounced increase in utilization between 2007–2012 and relatively constant utilization between 2012–2017. Given the timely correlation, the observed increase between 2007–2012 is believed to be a reform effect. PHC utilization patterns following the reform have been described to correlate well with expected health care needs based on sex and age [13]. Here, increased utilization was shown to be unevenly distributed between defined gross sex- and age groups. Sex differences in utilization were observed to decrease in the oldest age group while they increased in the youngest age group. Taking sex differences in relative utilization at baseline into account, these changes could be interpreted as shifts in gender equity.

By choosing a different approach to investigate socioeconomic and geographical equity in PHC utilization, this study gives nuance to previous findings on the subject. Reports have previously shown how the Patient Choice Reform has facilitated an uneven distribution of PCCs within and between regions, affecting geographical equity in a negative direction [13–15]. In Region Skåne most new PCCs following the reform have been established in the more densely populated southwestern part of the region [14]. Despite the uneven distribution of PCCs, GP visits remained pro-poor 2 years following the reform in the three largest regions (Stockholm, Västra Götaland and Skåne) [27]. In Region Skåne the number of consultations increased relatively more for individuals with high-income than with low income [13]. A previous study using a cohort from Region Skåne, identified men and women over 64 years and with an income above median to have increased their PHC utilization the most at follow-up 2 years post-reform [6]. Here, using a longer follow-up, the only trajectory-group with both increasing utilization and association with higher SES, was that of older men. Furthermore, decreasing utilization in older men was associated with lower SES. To the contrary, increasing levels of utilization in the majority of female groups was associated with lower SES, the association getting stronger with falling age and higher levels of utilization. Also, female groups with decreasing utilization were all associated with higher SES. These shifts in utilization can be interpreted as pro-rich for older men and pro-poor for women. To our knowledge, relationships between PHC utilization in women and SES have not previously been reported.

Increased geographical inequity in the allocation of PCCs could be a contributing factor to observed changes in utilization. With few exceptions, trajectory-groups with decreasing utilization were associated with residence in non-urban areas. Furthermore, all trajectory-groups where utilization increased to high levels were associated with residence in urban areas. These groups of becoming high utilizers were all female and associated with lower SES. A theoretically possible explanation for this could be increased health care needs in defined female population groups, and that improved accessibility following the Patient Choice Reform has led to increased equity in terms of utilization. This explanation seems unlikely given the relatively younger urban population (median 41 years) compared to the non-urban population (median 45 years). More likely, increased PHC utilization in urban areas is facilitated by an improved PHC accessibility with a lesser regard to actual health care needs. In support of this notion, there have been reports of change in health care seeking behavior among young adults since the reform, indicating a lower threshold for consulting health care professionals for minor health care issues [21, 28]. A contributing factor could be increased health care seeking behavior for mental health related issues. Such an increase would also provide insights to the marked sex difference in utilization between young women and men (utilization in younger women increased three-fold compared to men of the same age group). Increasing numbers of young adults, predominantly female, have been reported to experience mental health issues such as depression and anxiety [29–31]. Furthermore, PHC utilization for mental health issues has been shown to increase and diagnoses of depression and anxiety in PHC are twice as common in women as in men [31–33].

An explanation to the associations between the becoming high utilizers and low SES might also reside in differences in health literacy. Health literacy, referring to an individual’s capacity to meet complex demands of health in modern society [18], has likely become more important with the Patient Choice Reform. In urban areas, where the majority of new PCCs are located, patient choice is made more complex both due to the multitude of PCCs, but also due to the diversification of offered services. In addition to more traditional PCCs, the range of available services include drop-in units, out-of-hours clinics, nurse-led clinics, and diagnosis-specific health care services [34]. As inadequate health literacy is strongly associated with low SES [35], it likely serves as a contributing factor to the observed pattern of increasing utilization in urban areas. Health literacy might also be an explanation for the observed increase in utilization in older men associated with higher SES and residence in non-urban areas. Adequate health literacy for this group might serve as an enabling factor to navigate the choice-system and to gain access to PCCs. PCCs located in urban areas will likely require more means to access for individuals living in non-urban areas.

As proxies for high SES, one might expect higher income, higher educational level and being married or cohabitant to affect utilization in a similar fashion. Our results suggest income and civil status rather than education to be indicative for utilization in older individuals. Furthermore, income and education rather than civil status seemed indicative for utilization among younger and middle-aged individuals. These observations are likely due to changing availability and relevance of higher education along with changing social implications of marriage over time.

Strengths of this study are the cohort design, large study population, and reliable data sources. Compared to an ecological design, a closed cohort allows for analyses on changes in utilization over time on an individual level. By constructing a cohort, a constant population could be studied throughout a 10-year period and potential effects by fluctuations in the population due to new entries or exits could be disregarded. Consequently, other factors implicative for PHC utilization are likely to have changed during the observation period as well – both on the individual and population level – and inferences about the potential effect of the reform on utilization should be made with caution.

On the individual level, both income and education can vary over time, typically in early adulthood and following retirement. Sensitivity analyses defining income- and educational level at endpoint instead of at baseline showed little or no effect on outcomes. The data in this study are based on register information gathered for administrative purposes. In Sweden, there is a long tradition of recording, storing, and managing information in registers, both by individual regions and government authorities. Data validity in these registers is known to be high. In addition, the use of family income per consumption unit instead of individual income gives a more realistic estimation of an individual’s SES.

On the population level, health care utilization can be affected by a range of different factors such as the external environment (physical, political and economic), the organization of the health care system and population characteristics (predisposing characteristics, enabling resources and need) [36]. Throughout the study period, external factors have remained stable and are not believed to have influenced the results. However, during a 10-year follow-up, minor changes in specialized health care provision are likely to have taken place. Although minor, such changes might have impacted subgroups of the population and thus have affected PHC utilization in those subgroups. Furthermore, the increasing age of the population has probably impacted subgroups of the population to different degrees during the 10 year follow-up. Identified trajectory-groups are likely a representation of heterogeneity in utilization affected by the reform as well as heterogeneity in underlying health trajectories of individuals and, in turn, their individual care needs. Hence, a limitation of this study is the absence of control for morbidity and changes in diagnostic patterns over time. Considering the basis for an equitable health care – the appropriation of health care services according to differences in patient needs – analyses of inequities in health care utilization should be controlled for morbidity (as a proxy for ‘need’). The Johns Hopkins ACG (Adjusted Clinical Groups) Case-Mix System was developed to evaluate the relationship between individual morbidity and utilization and is a widely used tool when controlling for morbidity [37]. To evaluate the relationship between individual morbidity and utilization, the initial regression model in this study applied ACG as an independent variable. At the beginning and end of follow-up, all individuals were subscribed one of six Resource Utilization Bands (RUBs) – RUB 0 corresponding to no need for health care and RUB 5 a high need, each RUB consisting of individuals with the same type and degree of comorbidity. In this initial model, regardless of being defined at baseline or endpoint, ORs for RUB showed an absolute correlation to health care utilization. Adding no useful information, ACG was excluded in the final regression model. Since knowledge of potential changes in diagnostic patterns might have given useful insights to underlying causes of observed changes in utilization, absence of diagnostic information limits the study.

Another limitation to this study concerns loss of information due to inherent characteristics of the study method. A premise for analyzing differences in utilization trajectories, according to the method used here, is the categorization of utilization-groups based on the annual number of GP visits. This categorization implies a loss of information and, hence, could have affected the sensitivity of the results in a negative fashion. Due to the large study population, and the large amount of PHC utilization data, we do not believe this information loss sufficient to affect the final results.

In this study, only real-life visits were registered in the outcome variable. However, excluding virtual doctor visits is not believed to have a significant effect on reported outcomes. Online care platforms, making virtual doctor visits possible, just entered the Swedish PHC market in 2016. In 2017, virtual visits constituted only 2% of the total number of GP visits nationwide [28].

An essential limitation to this study is the used outcome measure. The measure of the number of GP visits over time showed nothing about the content or the quality of individual visits – if the patient has received the care needed or not. A fewer number of longer visits might be more beneficial to a patient than several shorter visits. A possible explanation for the observed increase in utilization in younger women might be a loss in quality in the health care given. For a better understanding, further studies using different measures and methodology are warranted. In this respect, potential changes in continuity of care, as a quality measure, would be relevant to investigate for groups of the population.

The results of this study can be generalized to other Swedish regions with some considerations – foremost regarding population demographics and regional variation in health care provision. In addition, regional reimbursement models are known to differ in terms of to which degree they are based on capitation or fee-for-service, and whether capitation is risk-adjusted or not. Regional variations in PHC reimbursement likely affect PHC utilization and comparisons between regions need to take such differences into consideration.

## Conclusions

This study showed changes in PHC utilization over a 10-year period overlapping the patient choice reform in the Swedish Region of Skåne. The observed increase in utilization in the general population was unevenly distributed between defined sex- and age groups. Sex differences in utilization decreased in the oldest age group while they increased in the youngest age group, the latter explained by a marked increase in utilization among younger women. Furthermore, the impact of socioeconomic and geographic determinants on PHC utilization varied in magnitude and direction between groups of the population. Increasing utilization among older men was associated with higher SES and in female groups associated with lower SES. Defined groups of becoming high utilizers were all female and were all associated with low SES and residence in urban areas, these associations getting stronger with falling age. Likely there are several plausible explanations behind the unequal distribution of PHC utilization. Possible contributing factors are changing health care seeking behavior among young adults and the increasing importance of health literacy in the patient choice system. To get a more complete understanding of how trends in PHC utilization affect equity in health care, further studies should also include quality aspects of PHC utilization, such as continuity of care, and equity aspects of secondary health care utilization.

### Supplementary Information

**Additional file 1: Additional Table 1.** Trajectory fit statistics. **Additional Table 2a.** Trajectory-group characteristics at baseline for males. Numbers indicate percent (%) of subgroup total if not otherwise specified. **Additional Table 2b.** Trajectory-group characteristics at baseline for females. Numbers indicate percent (%) of subgroup total if not otherwise specified. **Additional Table 3a.** Bivariate logistic regression on male trajectory-groups showing odds ratios (ORs) of change in utilization due to differences in predisposing and enabling factors. **Additional Table 3b.** Bivariate logistic regression on female trajectory-groups showing odds ratios (ORs) of change in utilization due to differences in predisposing and enabling factors.

**Additional file 2: Additional Figure 1.** Trajectory analysis datasets. Trajectory analysis output showing trajectories of primary health care utilization between 2007–2017. On the Y-axis, the number of annual GP visits per individual are categorized by 4 utilization-groups corresponding to; 0 visits, 1 visit, 2–3 visits, and more than 3 visits.

## Data Availability

The trajectory datasets generated and analyzed during this study are available from the corresponding author upon reasonable request.

## Abbreviations

GP: General practitioner
PCC: Primary health care center
PHC: Primary health care
SES: Socioeconomic status
