# Correction: Trajectories of primary health care utilization: a 10-year follow-up after the Swedish Patient Choice Reform of primary health care

**DOI:** 10.1186/s12913-024-10858-8

**Published:** 2024-03-18

**Authors:** Hannes Kohnke, Andrzej Zielinski, Anders Beckman, Henrik Ohlsson

**Affiliations:** 1 Department of Clinical Sciences, Faculty of Medicine, Lund University, Lund, Sweden; 2 Bräkne-Hoby Vårdcentral, Parkvägen 4, 370 10 Bräkne‑Hoby, Sweden; 3 Blekinge Centre of Competence, Region Blekinge, Vårdskolevägen 5, 371 41 Karlskrona, Sweden; 4 Department of Clinical Sciences, Center for Primary Health Care Research, Lund University Clinical Research Centre (CRC), Box 50332, 202 13 Malmö, Sweden

**Correction: BMC Health Services Research (2023) 23:1294**

**https://doi.org/10.1186/s12913-023-10326-9**

In Table 1 of this article, the data in the column ‘Male, 20-34’, rows ‘2007’, ‘2012’ and ‘2017’ were mistakenly listed under the column ‘Male, 55-69’, rows ‘2007’, ‘2012’ and ‘2017’ and vice versa.

In addition, the data in the column ‘Female, 20-34’, rows ‘2007’, ‘2012’ and ‘2017’ were mistakenly listed under the column ‘Female, 55-69’, rows ‘2007’, ‘2012’ and ‘2017’ and vice versa.

The incorrect and correct version of Table 1 can be found below and the article has been updated.

**Incorrect Table 1:**

**Table 1:** Cohort population characteristics and general trends in utilization in defined sex- and age groups between 2007 and 2017

Sex		Male			Female			
Age group (years)		20-34	35-54	55-69	20-34	35-54	55-69	Total
**Number of individuals (N)**		93,427	145,045	88,365	92,405	145,484	94,572	659,298
**Income (% of total in age group)**	low income	30.5	31.8	29.8	38.9	36.0	36.6	34.0
	medium income	30.3	33.4	34.0	35.1	32.7	32.7	33.0
	high income	39.2	34.8	36.2	26.0	31.3	30.7	33.0
**Education (% of total in age group)**	primary school	4.9	6.2	16.3	4.0	4.2	17.1	8.2
	secondary school	39.0	39.6	42.0	35.8	38.9	43.2	39.7
	higher education	56.1	54.2	41.6	60.3	57.0	39.7	52.2
**Civil status (% of total in age group)**	single	81.1	47.0	32.9	72.0	44.3	38.0	51.5
	married/cohabitant	18.9	53.0	67.1	28.0	55.7	62.0	48.5
**Municipality of residence (% of total in age group)**	urban	50.5	40.6	36.1	51.1	40.8	38.0	4.6
	semi-urban	25.0	30.5	31.9	25.0	30.5	31.4	29.3
	rural	24.5	28.9	32.0	23.9	28.7	30.5	28.2
**Mean number of annual GP visits per person**	2007	1.32	0.93	0.71	1.73	1.4	1.18	1.21
	2012	1.58	1.05	0.83	1.95	1.58	1.48	1.41
	2017	1.66	1.11	0.8	1.94	1.6	1.41	1.42
**Absolute increase in annual GP visits 2007 vs, 2017 (N)**		6,657	26,369	29,579	19,017	29,585	20,163	131,370
**Relative increase in annual GP visits 2007 vs, 2017 (%)**		11	20	25	20	15	12	17

**Correct Table 1:**

**Table 1:** Cohort population characteristics and general trends in utilization in defined sex- and age groups between 2007 and 2017

Sex		Male			Female			
**Age group (years)**		**20–34**	**35–54**	**55–69**	**20–34**	**35–54**	**55–69**	**Total**
**Number of individuals (N)**		93,427	145,045	88,365	92,405	145,484	94,572	659,298
**Income (% of total in age group)**	low income	30.5	31.8	29.8	38.9	36.0	36.6	34.0
	medium income	30.3	33.4	34.0	35.1	32.7	32.7	33.0
	high income	39.2	34.8	36.2	26.0	31.3	30.7	33.0
**Education (% of total in age group)**	primary school	4.9	6.2	16.3	4.0	4.2	17.1	8.2
	secondary school	39.0	39.6	42.0	35.8	38.9	43.2	39.7
	higher education	56.1	54.2	41.6	60.3	57.0	39.7	52.2
**Civil status (% of total in age group)**	single	81.1	47.0	32.9	72.0	44.3	38.0	51.5
	married/cohabitant	18.9	53.0	67.1	28.0	55.7	62.0	48.5
**Municipality of residence (% of total in age group)**	urban	50.5	40.6	36.1	51.1	40.8	38.0	4.6
	semi-urban	25.0	30.5	31.9	25.0	30.5	31.4	29.3
	rural	24.5	28.9	32.0	23.9	28.7	30.5	28.2
**Mean number of annual GP visits per person**	2007	0.71	0.93	1.32	1.18	1.4	1.73	1.21
	2012	0.83	1.05	1.58	1.48	1.58	1.95	1.41
	2017	0.8	1.11	1.66	1.41	1.6	1.94	1.42
**Absolute increase in annual GP visits 2007 vs, 2017 (N)**		6,657	26,369	29,579	19,017	29,585	20,163	131,370
**Relative increase in annual GP visits 2007 vs, 2017 (%)**		11	20	25	20	15	12	17

