# Supplementary material for: Trajectories of primary health care utilization: a 10-year follow-up after the Swedish Patient Choice Reform of primary health care
Source: BMC Health Serv Res. 2023 Nov 23;23:1294. doi: 10.1186/s12913-023-10326-9 (PMC10668480; doi:10.1186/s12913-023-10326-9)
Supplement: Supplementary file 1 — Additional file 1: Additional Table 1. Trajectory fit statistics. Additional Table 2a. Trajectory-group characteristics at baseline for males. Numbers indicate percent (%) of subgroup total if not otherwise specified. Additional Table 2b. Trajectory-group characteristics at baseline for females. Numbers indicate percent (%) of subgroup total if not otherwise specified. Additional Table 3a. Bivariate logistic regression on male trajectory-groups showing odds ratios (ORs) of change in utilization due to differences in predisposing and enabling factors. Additional Table 3b. Bivariate logistic regression on female trajectory-groups showing odds ratios (ORs) of change in utilization due to differences in predisposing and enabling factors. [file 12913_2023_10326_MOESM1_ESM.docx]

**Additional Table 1**. Trajectory fit statistics.

| Sex | Age group (years) | Number of trajectories | Number of datapoints | BIC | AIC | Log-likelihood ratio |
| --- | --- | --- | --- | --- | --- | --- |
| Males | **20-34** | 3 | 1,027,697 | -1,172,314 | -1,172,261 | -1,172,252 |
|  |  | 4 | 1,027,697 | -1,170,101 | -1,170,029 | -1,170,017 |
|  |  | 5* | 1,027,697 | -1,168,295 | -1,168,206 | -1,168,191 |
|  |  | 6 | 1,027,697 | -1,167,812 | -1,167,706 | -1,167,688 |
|  |  | 7 | 1,027,697 | -1,167,026 | -1,166,901 | -1,166,880 |
|  |  | 8 | 1,027,697 | -1,166,646 | -1,166,504 | -1,166,480 |
|  | **35-54** | 3 | 1,595,506 | -1,973,365 | -1,973,310 | -1,973,301 |
|  |  | 4 | 1,595,506 | -1,966,231 | -1,966,157 | -1,966,145 |
|  |  | 5 | 1,595,506 | -1,964,309 | -1,964,217 | -1,964,202 |
|  |  | 6* | 1,595,506 | -1,961,092 | -1,960,981 | -1,960,963 |
|  |  | 7 | 1,595,506 | -1,959,438 | -1,959,309 | -1,959,288 |
|  |  | 8 | 1,595,506 | -1,958,074 | -1957,926 | -1,957,902 |
|  | **55-69** | 3 | 972,015 | -1,347,069 | -1,347,016 | -1,347,007 |
|  |  | 4 | 972,015 | -1,339,399 | -1,339,329 | -1,339329 |
|  |  | 5 | 972,015 | -1,336,018 | -1,335,929 | -1,335,914 |
|  |  | 6* | 972,015 | -1,333,580 | -1,333,474 | -1,333,456 |
|  |  | 7 | 972,015 | -1,332,977 | -1,332,853 | -1,332,832 |
| Females | **20-34** | 3 | 1,016,455 | -1,379,365 | -1,379,311 | -1,379,302 |
|  |  | 4 | 1,016,455 | -1,375,023 | -1,374,952 | -1,374,940 |
|  |  | 5 | 1,016,455 | -1,373,052 | -1,372,963 | -1,372,948 |
|  |  | 6* | 1,016,455 | -1,371,522 | -1,371,416 | -1,371,398 |
|  |  | 7 | 1,016,455 | -1,370,305 | -1,370,181 | -1,370,160 |
|  |  | 8 | 1,016,455 | -1,369,811 | -1,369,669 | -1,369,645 |
|  | **35-54** | 3 | 1,600,357 | -2,178,800 | -2,178,745 | -2,178,736 |
|  |  | 4 | 1,600,357 | -2,168,477 | -2,168,403 | -2,168,391 |
|  |  | 5 | 1,600,357 | -2,165,498 | -2,165,406 | -2,165,391 |
|  |  | 6 | 1,600,357 | -2,162,092 | -2,161,981 | -2,161,963 |
|  |  | 7* | 1,600,357 | -2,158,952 | -2,158,823 | -2,158,802 |
|  |  | 8 | 1,600,357 | -2,158,562 | -2,158,415 | -2,158,395 |
|  | **55-69** | 3 | 1,040,303 | -1,467,984 | -1,467,930 | -1,467,921 |
|  |  | 4 | 1,040,303 | -1,458,314 | -1,458,243 | -1,458,231 |
|  |  | 5 | 1,040,303 | -1,454,993 | -1,454,905 | -1,454,890 |
|  |  | 6* | 1,040,303 | -1,452,628 | -1,452,521 | -1,452,503 |
|  |  | 7 | 1,040,303 | -1,451,245 | -1,451,121 | -1,451,100 |
| AIC = Akaike information criterion BIC =Bayesian information criterion * denotes model chosen for further analyses | | | | | | |

**Additional Table 2a**. Trajectory-group characteristics at baseline for males. Numbers indicate percent (%) of subgroup total if not otherwise specified.

| Age group (years) | | 20-34 | | | | | 35-54 | | | | | | 55-69 | | | | | |
| --- | --- | --- | --- | --- | --- | --- | --- | --- | --- | --- | --- | --- | --- | --- | --- | --- | --- | --- |
| Trajectory-group | | Group 1 | Group 2 | Group 3 | Group 4 | Group 5 | Group 1 | Group 2 | Group 3 | Group 4 | Group 5 | Group 6 | Group 1 | Group 2 | Group 3 | Group 4 | Group 5 | Group 6 |
| Age (mean) | | 27.2 | 27.7 | 27.3 | 28.3 | 27.4 | 43.8 | 43.7 | 43.3 | 45.5 | 44.7 | 46.1 | 60.3 | 60.4 | 61.6 | 60.9 | 62.2 | 62.5 |
| Income (% of subgroup total) | low income | 31.9 | 31.5 | 27.0 | 37.7 | 32.0 | 34.4 | 30.6 | 29.0 | 35.0 | 30.0 | 47.5 | 33.3 | 30.6 | 28.0 | 27.6 | 32.0 | 38.3 |
|  | medium income | 26.9 | 32.7 | 32.2 | 33.6 | 28.1 | 26.4 | 35.0 | 33.6 | 32.9 | 34.1 | 29.2 | 28.7 | 34.0 | 35.2 | 32.5 | 35.1 | 33.8 |
|  | high income | 41.1 | 35.6 | 40.7 | 28.6 | 39.8 | 39.1 | 34.3 | 37.2 | 32.0 | 35.8 | 23.2 | 37.8 | 35.2 | 36.6 | 39.8 | 32.7 | 27.8 |
| Education (% of subgroup total) | primary school | 3.9 | 6.2 | 4.1 | 10.7 | 4.7 | 7.2 | 5.3 | 5.1 | 7.6 | 6.5 | 10.6 | 15.3 | 16.2 | 16.2 | 14.8 | 17.7 | 20.4 |
|  | secondary school | 33.1 | 44.3 | 39.3 | 53.0 | 37.5 | 33.2 | 39.1 | 36.4 | 44.2 | 39.8 | 49.8 | 36.1 | 40.9 | 43.0 | 39.7 | 45.2 | 46.4 |
|  | higher education | 62.8 | 49.4 | 56.5 | 36.2 | 57.6 | 59.4 | 55.4 | 58.4 | 48.1 | 53.6 | 39.5 | 48.5 | 42.8 | 40.7 | 45.3 | 37.0 | 33.1 |
| Civil Status(% of subgroup total) | single | 84.2 | 78.1 | 80.9 | 73.6 | 81.9 | 54.0 | 45.0 | 48.0 | 45.7 | 47.4 | 47.4 | 41.2 | 35.4 | 30.3 | 34.2 | 30.9 | 34.1 |
|  | married/cohabitant | 15.7 | 21.8 | 19.0 | 26.3 | 18.0 | 45.9 | 54.9 | 51.9 | 54.2 | 52.5 | 52.5 | 58.7 | 64.5 | 69.6 | 65.7 | 69.0 | 65.8 |
| Municipality of residence (% of subgroup total) | urban | 54.1 | 47.9 | 48.0 | 45.3 | 53.4 | 47.6 | 38.5 | 41.0 | 40.1 | 40.4 | 46.2 | 41.9 | 34.8 | 33.8 | 38.4 | 36.5 | 40.8 |
|  | semi-urban | 22.7 | 26.6 | 26.3 | 28.5 | 23.5 | 27.5 | 31.6 | 29.8 | 31.4 | 30.0 | 28.9 | 28.0 | 29.9 | 33.5 | 28.6 | 34.1 | 32.4 |
|  | rural | 23.1 | 25.3 | 25.5 | 26.0 | 23.0 | 24.8 | 29.8 | 29.1 | 28.4 | 29.5 | 24.7 | 29.9 | 35.2 | 32.5 | 32.9 | 29.3 | 26.6 |

**Additional Table 2b**. Trajectory-group characteristics at baseline for females. Numbers indicate percent (%) of subgroup total if not otherwise specified.

| Age group (years) | | 20-34 | | | | | | 35-54 | | | | | | | 55-69 | | | | | |
| --- | --- | --- | --- | --- | --- | --- | --- | --- | --- | --- | --- | --- | --- | --- | --- | --- | --- | --- | --- | --- |
| Trajectory-group | | Group 1 | Group 2 | Group 3 | Group 4 | Group 5 | Group 6 | Group 1 | Group 2 | Group 3 | Group 4 | Group 5 | Group 6 | Group 7 | Group 1 | Group 2 | Group 3 | Group 4 | Group 5 | Group 6 |
| Age (mean) | | 27.3 | 27.4 | 27.5 | 27.6 | 27.2 | 28.1 | 43.8 | 44.5 | 44.1 | 43.3 | 44.7 | 45.2 | 45.3 | 60.5 | 61.0 | 61.8 | 61.6 | 62.4 | 62.4 |
| Income (% of subgroup total) | low income | 38.9 | 48.9 | 34.9 | 37.9 | 39.6 | 49.1 | 31.9 | 35.4 | 32.3 | 32.1 | 45.4 | 44.5 | 54.5 | 36.3 | 32.7 | 40.0 | 35.7 | 41.5 | 48.7 |
|  | medium income | 31.9 | 32.6 | 35.9 | 38.2 | 33.7 | 34.8 | 31.8 | 33.4 | 32.8 | 34.2 | 30.7 | 31.0 | 26.9 | 30.1 | 33.1 | 33.1 | 33.3 | 31.5 | 29.7 |
|  | high income | 29.1 | 18.3 | 29.1 | 23.8 | 26.6 | 16.0 | 36.1 | 31.0 | 34.8 | 33.5 | 23.7 | 24.3 | 18.4 | 33.4 | 34.0 | 26.7 | 30.8 | 26.8 | 21.5 |
| Education (% of subgroup total) | primary school | 2.9 | 7.1 | 2.7 | 4.4 | 3.7 | 8.4 | 3.5 | 4.1 | 3.3 | 3.0 | 5.9 | 5.9 | 8.7 | 16.3 | 15.4 | 18.6 | 16.9 | 18.4 | 22.0 |
|  | secondary school | 28.3 | 44.9 | 32.9 | 40.7 | 33.1 | 51.2 | 32.2 | 39.7 | 35.7 | 35.5 | 45.9 | 46.7 | 50.8 | 38.8 | 40.6 | 46.4 | 43.6 | 45.1 | 48.6 |
|  | higher education | 68.7 | 47.9 | 64.2 | 54.7 | 63.1 | 40.3 | 64.1 | 56.0 | 60.9 | 61.4 | 48.0 | 47.3 | 40.3 | 44.7 | 43.9 | 34.8 | 39.3 | 36.3 | 29.2 |
| Civil Status(% of subgroup total) | single | 74.7 | 69.0 | 72.5 | 70.3 | 73.1 | 64.9 | 43.8 | 44.1 | 43.5 | 42.8 | 47.0 | 45.8 | 48.2 | 40.6 | 36.4 | 38.8 | 37.0 | 40.9 | 42.6 |
|  | married/cohabitant | 25.2 | 30.9 | 27.4 | 29.6 | 26.8 | 35.0 | 56.1 | 55.8 | 56.4 | 57.1 | 52.9 | 54.1 | 51.7 | 59.3 | 63.5 | 61.1 | 62.9 | 59.0 | 57.3 |
| Municipality of residence (% of subgroup total) | urban | 53.9 | 52.4 | 49.8 | 48.3 | 53.9 | 46.6 | 40.9 | 40.1 | 40.3 | 39.1 | 43.3 | 42.9 | 48.3 | 40.0 | 37.3 | 38.4 | 37.2 | 40.5 | 40.4 |
|  | semi-urban | 22.7 | 24.1 | 25.4 | 27.3 | 23.1 | 27.8 | 29.7 | 31.1 | 30.0 | 31.0 | 29.1 | 30.9 | 27.4 | 28.1 | 29.6 | 33.7 | 32.3 | 31.0 | 32.2 |
|  | rural | 23.3 | 23.4 | 24.6 | 24.3 | 22.8 | 25.4 | 29.2 | 28.7 | 29.6 | 29.7 | 27.5 | 26.0 | 24.1 | 31.7 | 32.9 | 27.8 | 30.3 | 28.4 | 27.2 |

**Additional Table 3a**. Bivariate logistic regression on male trajectory-groups showing odds ratios (ORs) of change in utilization due to differences in predisposing and enabling factors

| Age group (years) | | 20-34 | | 35-54 | | 55-69 | |
| --- | --- | --- | --- | --- | --- | --- | --- |
| Compared trajectory-groups* | | **Group 5 vs. 1 (ref.)** | **Group 3 vs. 2 (ref.)** | **Group 5 vs. 3 (ref.)** | **Group 2 vs. 4 (ref.)** | **Group 4 vs. 1 (ref.)** | **Group 2 vs. 3 (ref.)** |
| Corresponding generic trajectory-groups** | | **I vs. V (ref.)** | **III vs. VI (ref.)** | **I vs. V (ref.)** | **III vs. VI (ref.)** | **I vs. V (ref.)** | **III vs. VI (ref.)** |
| Analyzed utilization change | | **OR (CI) for increase from low utilization** | **OR (CI) for decrease from intermediate utilization** | **OR (CI) for increase from low utilization** | **OR (CI) for decrease from intermediate utilization** | **OR (CI) for increase from low utilization** | **OR (CI) for decrease from intermediate utilization** |
| Age | | 1.01 (1.00-1.01) | 0.98 (0.98-0.98) | 1.05 (1.04-1.05) | 0.95 (0.95-0.95) | 1.04 (1.03-1.05) | 0.93 (0.92-0.93) |
| Income | low income | 1 | 1 | 1 | 1 | 1 | 1 |
|  | middle income | 1.04 (0.99-1.10) | 1.15 (1.10-1.20) | 0.98 (0.94-1.02) | 1.22 (1.17-1.26) | 1.36 (1.27-1.46) | 0.88 (0.84-0.93) |
|  | high income | 0.97 (0.92-1.01) | 1.34 (1.28-1.40) | 0.93 (0.90-0.97) | 1.22 (1.18-1.27) | 1.27 (1.19-1.35) | 0.88 (0.83-0.93) |
| Education | primary school | 1 | 1 | 1 | 1 | 1 | 1 |
|  | secondary school | 0.94 (0.85-1.04) | 1.34 (1.23-1.46) | 0.85 (0.79-0.92) | 1.26 (1.18-1.34) | 1.13 (1.04-1.23) | 0.95 (0.89-1.01) |
|  | higher education | 0.76 (0.69-0.84) | 1.73 (1.59-1.88) | 0.71 (0.67-0.77) | 1.64 (1.53-1.74) | 0.96 (0.89-1.04) | 1.05 (0.99-1.12) |
| Civil status | single | 1 | 1 | 1 | 1 | 1 | 1 |
|  | married or cohabitant | 1.18 (1.12-1.24) | 0.84 (0.81-0.88) | 1.02 (0.99-1.06) | 1.03 (1.00-1.06) | 1.35 (1.28-1.42) | 0.79 (0.76-0.83) |
| Municipality of residence | urban | 1 | 1 | 1 | 1 | 1 | 1 |
|  | semi-urban | 1.05 (1.00-1.10) | 0.99 (0.95-1.03) | 1.02 (0.98-1.06) | 1.05 (1.01-1.09) | 1.11 (1.04-1.19) | 0.87 (0.82-0.91) |
|  | rural | 1.01 (0.96-1.06) | 1.01 (0.96-1.05) | 1.03 (0.99-1.07) | 1.09 (1.05-1.13) | 1.2 (1.13-1.28) | 1.05 (1.00-1.10) |
| OR = odds ratio CI = confidence interval * as shown in Additional Figure 1 ** as shown in Figure 1 | | | | | | | |

**Additional Table 3b**. Bivariate logistic regression on female trajectory-groups showing odds ratios (ORs) of change in utilization due to differences in predisposing and enabling factors

| Age group (years) | | 20-34 | | | | 35-54 | | | | 55-69 | |
| --- | --- | --- | --- | --- | --- | --- | --- | --- | --- | --- | --- |
| Compared trajectory-groups* | | **Group 5 vs. 1 (ref.)** | **Group 5 vs. 3 (ref.)** | **Group 2 vs. 3 (ref.)** | **Group 2 vs. 4 (ref.)** | **Group 3 vs. 1 (ref.)** | **Group 5 vs. 2 (ref.)** | **Group 6 vs. 7 (ref.)** | **Group 4 vs. 2 (ref.)** | **Group 5 vs. 4 (ref.)** | **Group 3 vs. 6 (ref.)** |
| Corresponding generic trajectory-groups** | | **II vs. VI (ref.)** | **II vs. VI (ref.)** | **II vs. VI (ref.)** | **II vs. VI (ref.)** | **I vs. V (ref.)** | **II vs. VI (ref.)** | **IV vs. VII (ref.)** | **III vs. VI (ref.)** | **II vs. VI (ref.)** | **IV vs. VII (ref.)** |
| Analyzed utilization change | | **OR (CI) for increase from low utilization** | **OR (CI) for increase from low utilization** | **OR (CI) for increase from intermediate utilization** | **OR (CI) for increase from intermediate utilization** | **OR (CI) for increase from low utilization** | **OR (CI) for increase from intermediate utilization** | **OR (CI) for decrease from high utilization** | **OR (CI) for decrease from intermediate utilization** | **OR (CI) for increase from intermediate utilization** | **OR (CI) for decrease from high utilization** |
| Age | | 0.99 (0.99-1.00) | 0.98 (0.98-0.99) | 1.00 (0.99-1.00) | 0.99 (0.98-1.00) | 1.01 (1.01-1.01) | 1.01 (1.00-1.01) | 1.00 (0.99-1.00) | 0.97 (0.96-0.97) | 1.05 (1.04-1.05) | 0.97 (0.96-0.97) |
| Income | low income | 1 | 1 | 1 | 1 | 1 | 1 | 1 | 1 | 1 | 1 |
|  | middle income | 1.04 (0.99-1.09) | 0.83 (0.79-0.87) | 0.65 (0.61-0.70) | 0.66 (0.62-0.71) | 1.01 (0.97-1.07) | 0.72 (0.68-0.76) | 1.41 (1.31-1.53) | 1.13 (1.09-1.17) | 0.81 (0.76-0.87) | 1.36 (1.26-1.47) |
|  | high income | 0.90 (0.85-0.94) | 0.81 (0.77-0.85) | 0.45 (0.42-0.49) | 0.60 (0.55-0.65) | 0.95 (0.90-0.99) | 0.60 (0.56-0.63) | 1.61 (1.48-1.75) | 1.19 (1.15-1.24) | 0.75 (0.70-0.80) | 1.51 (1.38-1.65) |
| Education | primary school | 1 | 1 | 1 | 1 | 1 | 1 | 1 | 1 | 1 | 1 |
|  | secondary school | 0.92 (0.81-1.03) | 0.76 (0.68-0.85) | 0.53 (0.47-0.61) | 0.69 (0.61-0.79) | 1.18 (1.06-1.32) | 0.80 (0.72-0.89) | 1.37 (1.21-1.55) | 1.22 (1.12-1.33) | 0.95 (0.88-1.03) | 1.13 (1.03-1.24) |
|  | higher education | 0.72 (0.64-0.81) | 0.74 (0.66-0.82) | 0.29 (0.25-0.33) | 0.55 (0.49-0.62) | 1.02 (0.91-1.13) | 0.59 (0.54-0.66) | 1.74 (1.54-1.98) | 1.50 (1.38-1.64) | 0.85 (0.79-0.92) | 1.41 (1.28-1.55) |
| Civil status | single | 1 | 1 | 1 | 1 | 1 | 1 | 1 | 1 | 1 | 1 |
|  | married or cohabitant | 1.09 (1.04-1.14) | 0.97 (0.93-1.01) | 1.19 (1.11-1.27) | 1.07 (1.00-1.14) | 1.02 (0.98-1.06) | 0.89 (0.85-0.93) | 1.10 (1.03-1.17) | 1.05 (1.02-1.08) | 0.85 (0.80-0.90) | 1.17 (1.09-1.25) |
| Municipality of residence | urban | 1 | 1 | 1 | 1 | 1 | 1 | 1 | 1 | 1 | 1 |
|  | semi-urban | 1.02 (0.97-1.07) | 0.84 (0.80-0.88) | 0.90 (0.83-0.97) | 0.81 (0.76-0.88) | 1.03 (0.98-1.08) | 0.87 (0.82-0.92) | 1.27 (1.18-1.38) | 1.02 (0.98-1.06) | 0.88 (0.82-0.94) | 1.10 (1.01-1.19) |
|  | rural | 0.98 (0.93-1.03) | 0.86 (0.82-0.90) | 0.90 (0.84-0.97) | 0.89 (0.83-0.96) | 1.03 (0.98-1.08) | 0.89 (0.84-0.94) | 1.22 (1.12-1.32) | 1.06 (1.02-1.10) | 0.86 (0.80-0.92) | 1.08 (0.99-1.17) |
| OR = odds ratio CI = confidence interval * as shown in Additional Figure 1 ** as shown in Figure 1 | | | | | | | | | | | |
