# Supplementary figures and images for: Trajectories of primary health care utilization: a 10-year follow-up after the Swedish Patient Choice Reform of primary health care
Source: BMC Health Serv Res. 2023 Nov 23;23:1294. doi: 10.1186/s12913-023-10326-9 (PMC10668480; doi:10.1186/s12913-023-10326-9)

**a.** Men 55-69 years **b.** Women 55-69 years


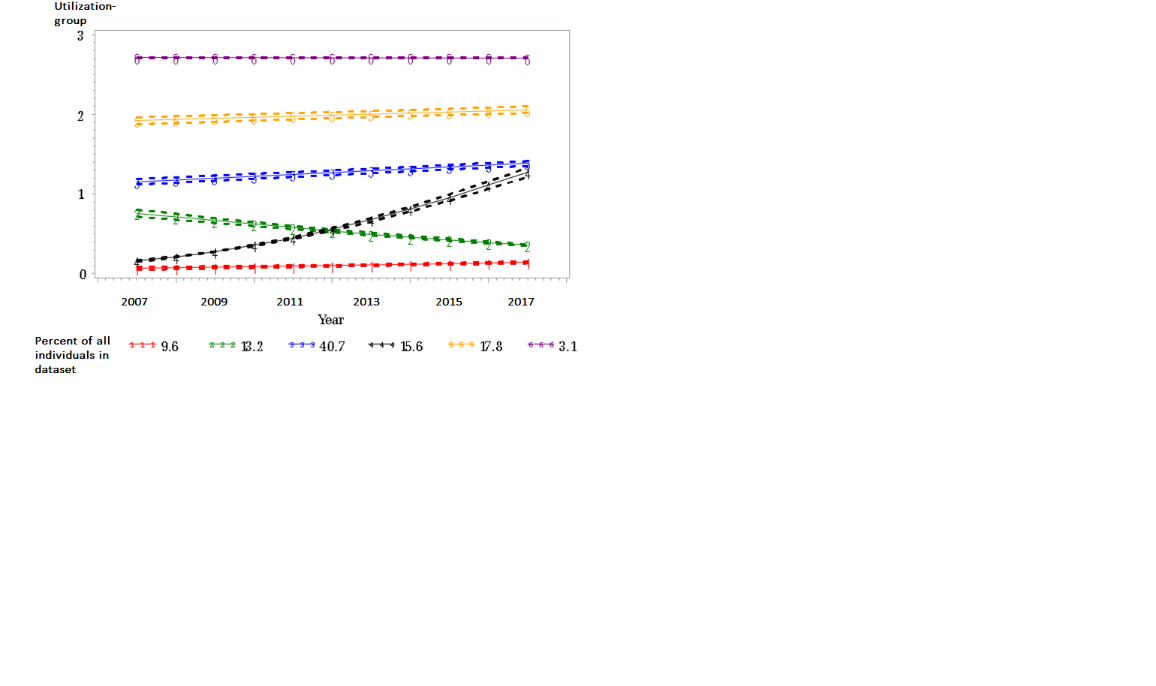

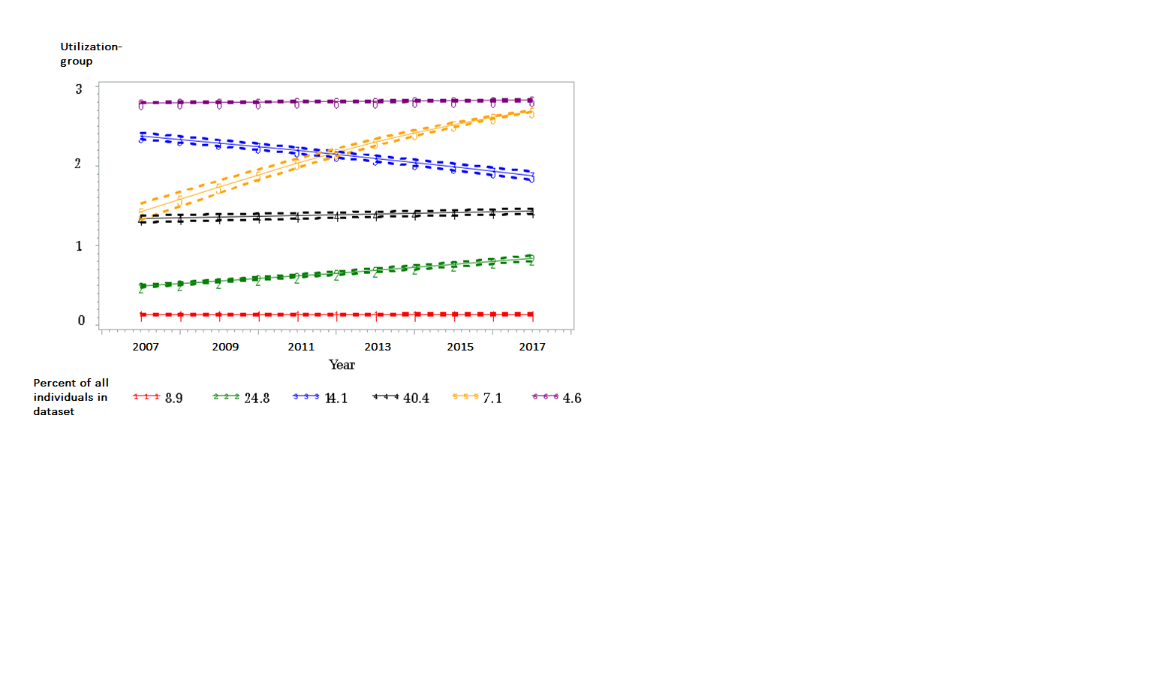


**c.** Men 35-54 years **d.** Women 35-54 years


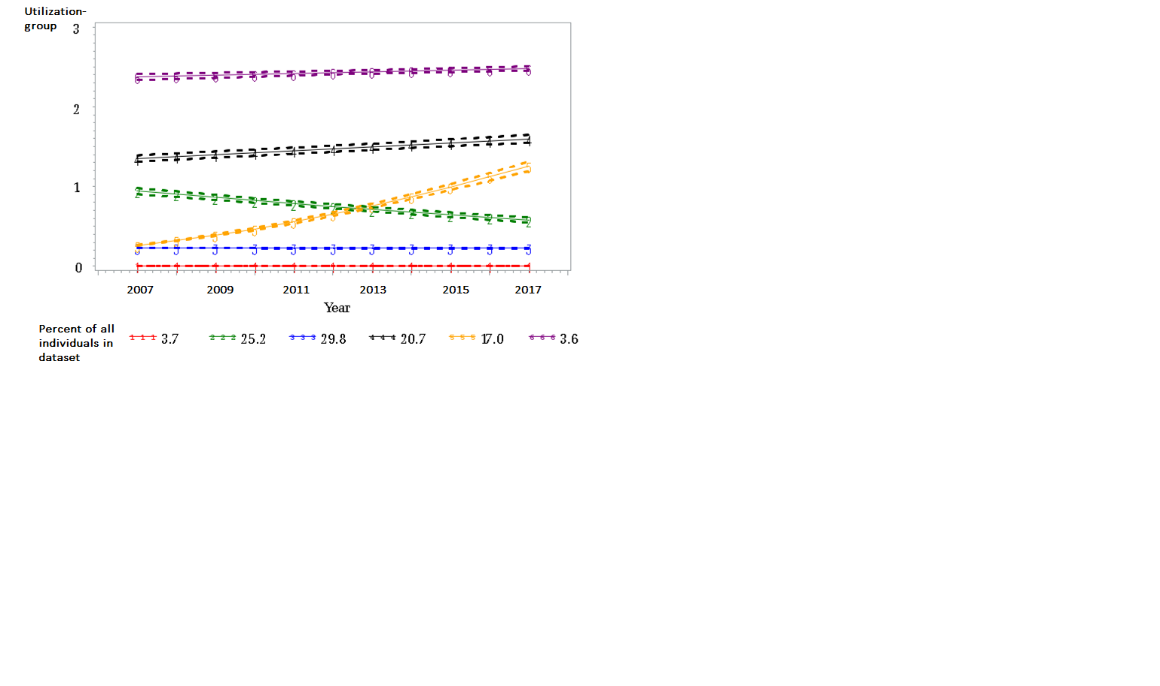

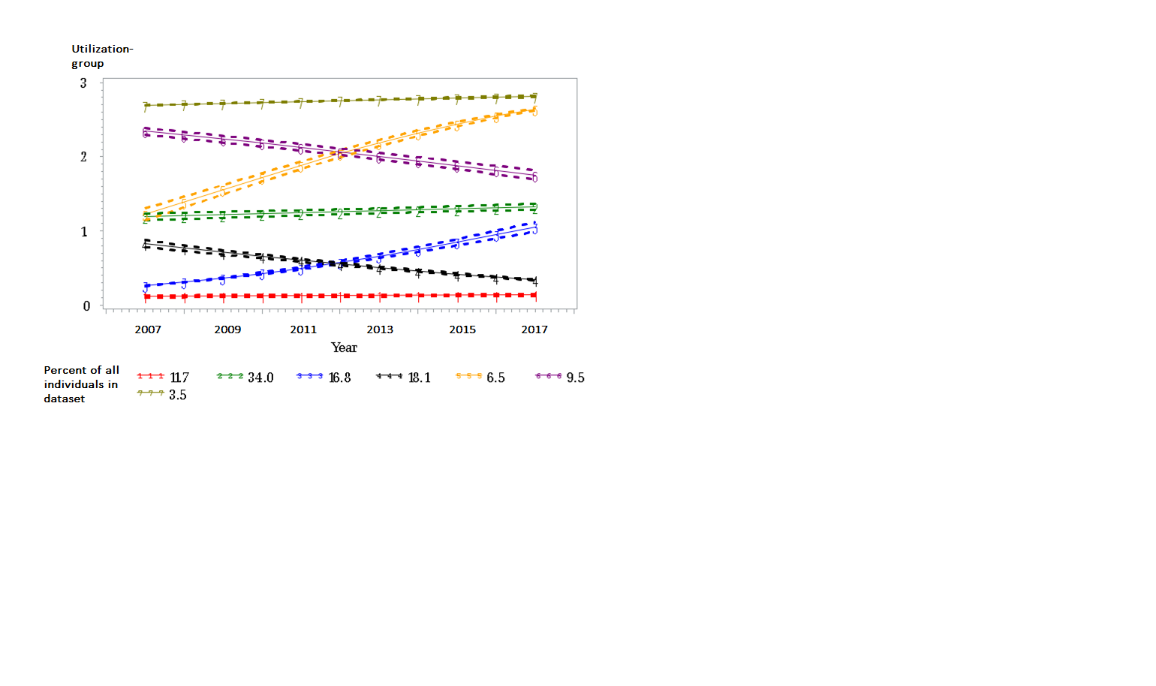


**e.** Men 20-34 years **f.** Women 20-34 years


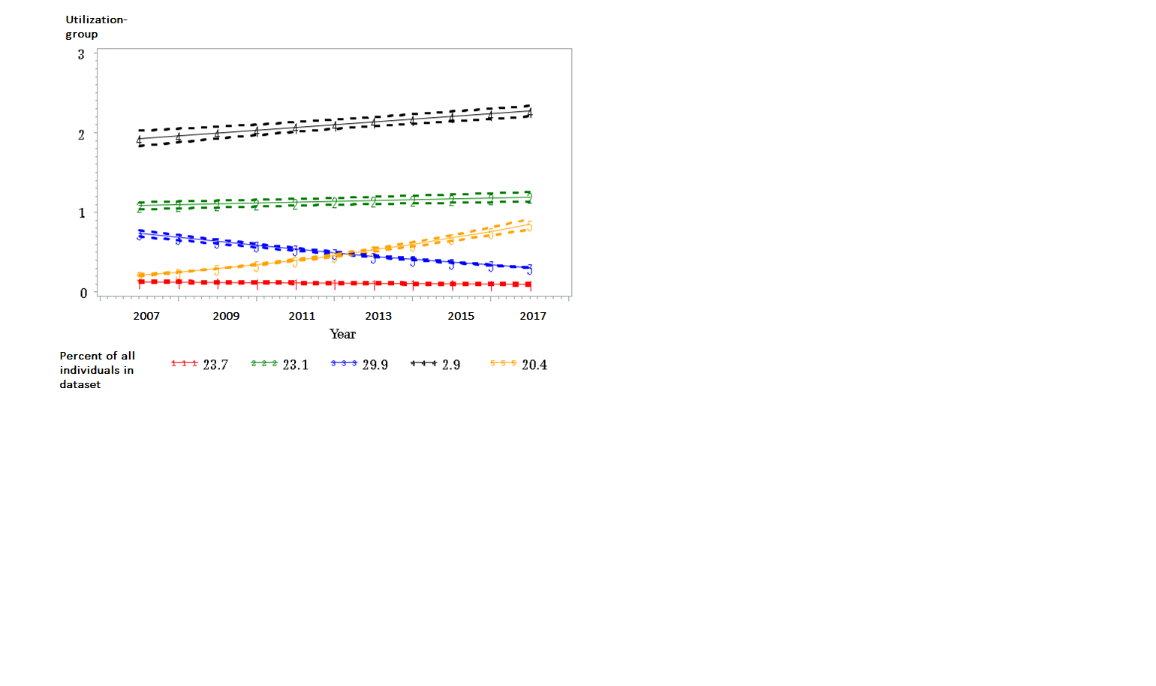

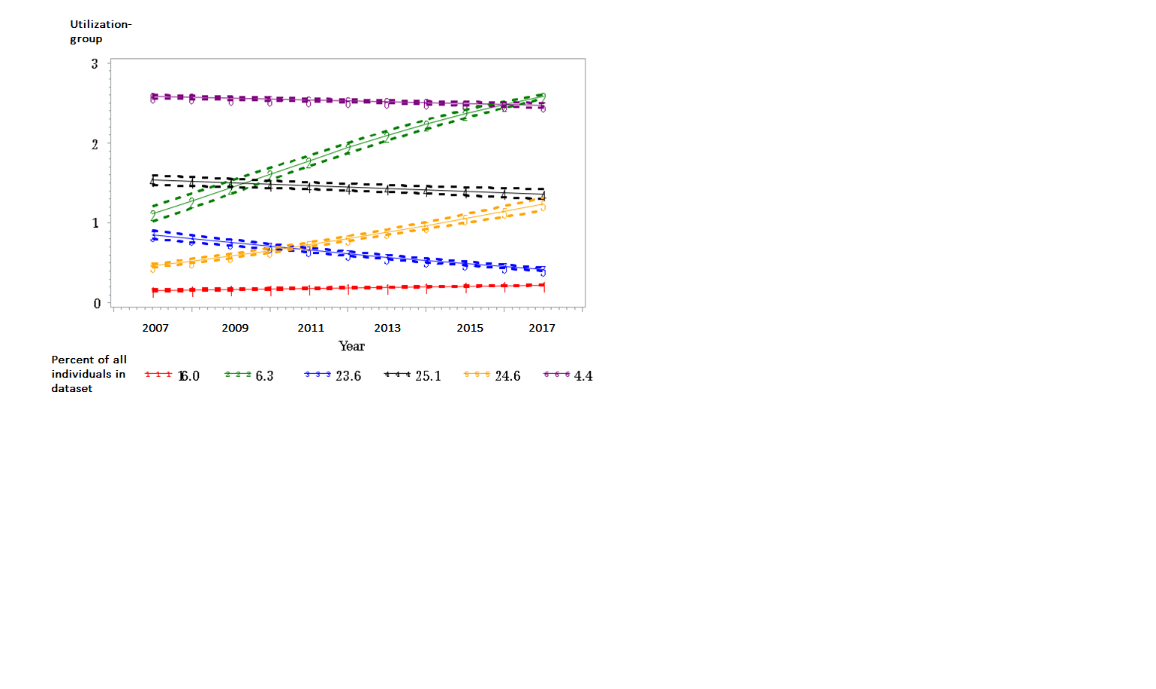


**2**

**4**

**3**

**1**

**2**

**4**

**3**

**1**

**2**

**4**

**3**

**1**

**2**

**4**

**3**

**1**

**2**

**4**

**3**

**1**

**2**

**4**

**3**

**1**

Supplement: Supplementary file 2 — Additional file 2: Additional Figure 1. Trajectory analysis datasets. Trajectory analysis output showing trajectories of primary health care utilization between 2007–2017. On the Y-axis, the number of annual GP visits per individual are categorized by 4 utilization-groups corresponding to; 0 visits, 1 visit, 2–3 visits, and more than 3 visits. [file 12913_2023_10326_MOESM2_ESM.docx]
